# Patient skin dose measurements using a cable free system MOSFETs based in fluoroscopically guided percutaneous vertebroplasty, percutaneous disc decompression, radiofrequency medial branch neurolysis, and endovascular critical limb ischemia

**DOI:** 10.1120/jacmp.v16i1.5020

**Published:** 2015-01-08

**Authors:** Maria D. Falco, Salvatore Masala, Matteo Stefanini, Roberto Fiori, Roberto Gandini, Paolo Bagalà, Daniele Morosetti, Eros Calabria, Alessia Tonnetti, Gianluca Verona‐Rinati, Riccardo Santoni, Giovanni Simonetti

**Affiliations:** ^1^ Department of Diagnostic Imaging Molecular Imaging, Interventional Radiology and Radiotherapy, University of Rome Tor Vergata Rome; ^2^ INFN–Department of Industrial Engineering University of Rome Tor Vergata Rome Italy

**Keywords:** OneDose MOSFET system, maximum skin dose, dose‐area product, fluoroscopically guided interventions, reference levels

## Abstract

The purpose of this work has been to dosimetrically investigate four fluoroscopically guided interventions: the percutaneous vertebroplasty (PVP), the percutaneous disc decompression (PDD), the radiofrequency medial branch neurolysis (RF) (hereafter named spine procedures), and the endovascular treatment for the critical limb ischemia (CLI). The X‐ray equipment used was a Philips Integris Allura Xper FD20 imaging system provided with a dose‐area product (DAP) meter. The parameters investigated were: maximum skin dose (MSD), air kerma (Ka,r), DAP, and fluoroscopy time (FT). In order to measure the maximum skin dose, we employed a system based on MOSFET detectors. Before using the system on patients, a calibration factor $F_{c}$ and correction factors for energy ($C_{\text{kV}}$) and field size ($C_{\text{FD}}$) dependence were determined. Ka,r, DAP, and FT were extrapolated from the X‐ray equipment. The analysis was carried out on 40 patients, 10 for each procedure. The average fluoroscopy time and DAP values were compared with the reference levels (RLs) proposed in literature. Finally, the correlations between MSD, FT, Ka,r, and DAP values, as well as between DAP and FT values, were studied in terms of Pearson's product‐moment coefficients for spine procedures only. An $F_{c}$ value of 0.20 and a very low dependence of $C_{\text{FD}}$ on field size were found. A third‐order polynomial function was chosen for $C_{\text{kV}}$. The mean values of MSD ranged from 2.3 to 10.8 cGy for CLI and PVP, respectively. For these procedures, the DAP and FT values were within the proposed RL values. The statistical analysis showed little correlation between the investigated parameters. The interventional procedures investigated were found to be both safe with regard to deterministic effects and optimized for stochastic ones. In the spine procedures, the observed correlations indicated that the estimation of MSD from Ka,r or DAP was not accurate and a direct measure of MSD is therefore recommended.

PACS number: 87

## I. INTRODUCTION

In the past twenty years, the clinical practice of fluoroscopically guided interventions (FGIs) that use image guidance has been greatly improved. FGIs are less intrusive with respect to surgery and their benefits are clear: high safety, accuracy, and low morbidity.^(1)^ However, some procedures use either long fluoroscopy times or high numbers of frames, exposing the patients to relevant doses which increase the probability of stochastic effects and can, in extreme cases, exceed the threshold of deterministic ones.

To promote the management of patient doses and to avoid unnecessary stochastic radiation risks the diagnostic reference levels (DRL)^2^, ^3^, ^4^, ^5^ can be used. However, although several international commissions^4^, ^6^, ^7^ seriously recommend the radiation protection of the patient in terms of DRL, at present the DRL values proposed by international protocols refer principally to standard radiology or CT scan. Regarding interventional procedures, only few references in the scientific literature suggest reference levels (RL) for some FGIs.^8^, ^9^, ^10^ In literature, the RLs are stated in terms of maximum skin dose (MSD) and dose‐area product (DAP), but also other parameters, such as fluoroscopy time, number of frames or effective dose, are typically used.^2^, ^11^, ^12^

Deterministic effects may occur days or months after an acute radiation exposure if the dose exceeds a threshold, and their intensity is directly proportional to the dose. In our case, the critical organ is the skin and the severity of the effect itself is related to the maximum skin dose (MSD), which should therefore be evaluated.^(13)^ If the MSD value exceeds the 2 Gy deterministic threshold, skin injuries will arise, such as transient erythema.

While in modern X‐ray systems DAP can be directly measured by means of a built‐in dosimeter, MSD is a more complex quantity to estimate. The main difficulty in measuring the MSD is to know the point where the dose attains its maximum value, which is *a priori* unknown. Some methods use large thermoluminescent dosimeter (TLD) detector arrays, large‐area slow films, including the self‐developing GAFCHROMIC films, in order to calculate the MSD from the corresponding dose distributions. However, the complexity of the calibration procedure and/or the long processing time makes them unpractical for routine use. To simplify the measurements, some authors used point detectors (TLDs, solid‐state active dosimeters such as silicon diodes or metal oxide semiconductor field‐effect transistors (MOSFETs)), properly positioned in the most irradiated anatomical region.^14^, ^15^, ^16^ The advantages and disadvantages of these *in vivo* dosimeters are well described in literature.^(17)^

The aim of this work is the dosimetric investigation of four FGIs: the percutaneous vertebroplasty (PVP), the percutaneous disc decompression (PDD), the radiofrequency medial branch neurolysis (RF) (hereafter named spine procedures), and the endovascular treatment for the critical limb ischemia (CLI). The procedures investigated in this work were chosen as few data can be found in the literature concerning both delivered skin dose and RL values. Furthermore, given that our department is a national reference center for these interventions, a direct skin dose measurement on patients has become mandatory in order to confirm the good practice of the operators.

## II. MATERIALS AND METHODS

### A. Calibration in phantom

The X‐ray equipment used in this study was a Philips Integris Allura Xper FD20 (Philips Medical Systems DMC GmbH, Hamburg, Germany). The source‐to‐image receptor distance (SID) ranged from 90 to 120 cm. The tube voltage and current settings were controlled by an automatic exposure control. The installed DAP meter was calibrated according to protocols available in literature.^2^, ^18^, ^19^, ^20^, ^21^, ^22^ The kilovolt, current, and current‐time product accuracy and total tube filtration of the angiographic unit were annually measured as part of quality assurance program.

**Figure 1:**
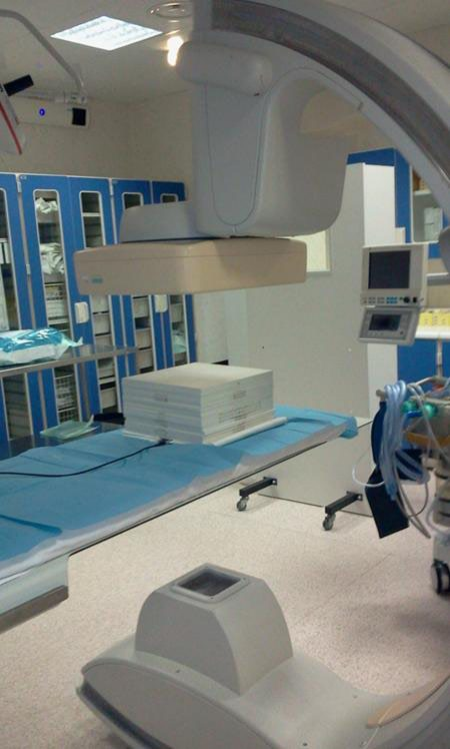
X‐ray equipment in the angiographic room for the in phantom calibration in the reference setting: tube rotation angle at 180°, SSD on the couch of 50 cm (couch thickness 9 cm), and SID 120 cm.

Dose measurements were performed using the OneDose (Sicel Technologies, Morrisville, NC) system based on p‐type MOSFET detectors, originally calibrated for radiotherapy. The details of this system, its use in radiotherapy, and some preliminary results in radiology, have been reported in literature.^16^, ^23^, ^24^, ^25^, ^26^, ^27^, ^28^, ^29^ The dosimeters are cable free and provided with an adhesive backing to be attached to the patient skin. Since the OneDose system is calibrated in the MV energy range, to use it in the kV energy range, a calibration factor $F_{c}$ and correction factors for energy and field of view dependence ($C_{\text{kV}}$ and $C_{\text{FD}}$) were determined, following the procedures reported in literature.^(29)^ The reference setting (RS) for $F_{c}$ determination was chosen as follows: tube rotation angle at 180°, tube voltage 80 k V, current‐time product 50 mAs; diagonal of the field‐of‐view (FD) 48 cm, source to skin distance (SSD) on the couch 50 cm (couch thickness 9 cm), and SID 120 cm. The energy dependence correction factor $C_{\text{kV}}$ was determined at the energies of 60 kV, 70 kV, 80 kV, 100 kV, and 120 kV. The $C_{\text{FD}}$ was obtained in the 22 cm, 31 cm, and 48 cm FD values. As the FD depends on SID, we determined correction factor $C_{\text{FD}}$ for a SID of 90 cm, as well. The values of the different correction factors for energies and field sizes were extrapolated by using proper best‐fit functions. As the investigated techniques employed two variable additional filters (0.4 mm Cu+1.0 mm Al for fluoroscopy mode or 0.1 mm Cu+1.0 mm Al for radiographic mode), we repeated the entire calibration process in both conditions. The detector readings were compared to the reference doses measured with a solid‐state detector, UNFORS XI (RaySafe Xi R/F Detector; Billdal, Sweden), calibrated at a Secondary Standard Dosimetry Laboratory. In all measurements, both dosimeters were positioned on the top of the table at a distance of 4 cm from one another. A 15 cm thick slab of polymethyl methacrylate $\left(\rho =1.2\;g/{\text{cm}}^{3}\right)$ was added on the table to provide the appropriate backscatter (Fig. 1). To ensure that the entire calibration procedure was independent from the dose, the linearity of the MOSFET readings as a function of the dose was also verified.

The GAFCHROMIC XRRV3 films (International Specialty Products, Wayne, NJ) have been used as an independent system to test the whole procedure. The dose response curve, which correlates the grey levels to the dose levels, was determined following the procedure reported in literature.^(29)^ The entrance surface doses in phantom obtained from GAFCHROMIC films were compared to those measured with the One Dose system and the solid‐state detector.

### B. Patient dose study

The present survey was a prospective randomized study including both male and female patients aged over 50. These patients complied with the inclusion criteria in the routine clinical practice for the accounted procedures. A total of 40 patients were recruited, 10 patients for each procedure. This study was approved by the ethical committee of the hospital and carried out after the informed consent had been given by the patients.

For the spine procedures, the patient was in a prone position on the couch. Two MOSFETs were attached in correspondence of each treated vertebral level, one underneath the patient for the antero–posterior projection (AP) and the other laterally for the latero–lateral projection (LL). In the CLI treatment the patient was supine. Eight MOSFETs were used, two placed on the proximal third femur (${\text{CLI}}_{\text{PF}}$), two on the distal third femur (${\text{CLI}}_{\text{DF}}$) close to the knee, two on the proximal third tibia (${\text{CLI}}_{\text{PT}}$), and the last two on the lower part of the ankle (${\text{CLI}}_{A}$).

For each procedure, a specific protocol was imposed by the radiologist. A set of data such as DAP value, rotation and tilt angle of the X‐ray tube, current and tube voltage for each rotation or tilt angle, cumulative fluoroscopy time (FT), and number of radiographic images were recorded for each patient. During the procedure, for every fluoroscopy time interval and for every radiography image, we recorded the corresponding $\Delta t_{i}$, ${\text{kV}}_{i},{\text{mA}}_{i}$, and ${\text{FD}}_{i}$ values, and calculated the corresponding correction factor $C_{\text{kVi}}$ and $C_{\text{FDi}}$. The final correction factors $C_{\text{kV}}$ and $C_{\text{FD}}$ were determined as:

(1) $$C_{kV}=\sum_{i}w_{i}C_{kVi}$$

and

(2) $$C_{FD}=\sum_{i}w_{i}C_{FDi}$$

where

(3) $$w_{i}=\Delta t_{i}mA_{i}/\sum \Delta t_{i}mA_{i}$$

is the time‐current weight for the ith irradiation condition.

The MSD, defined as the local skin dose at the point of its highest value, including the backscattering contribution, was finally determined by multiplying the MOSFET reading $M_{\text{MF}}$ for $F_{c},C_{\text{kV}}$, and $C_{\text{FD}}$:

(4) $$MSD(kV,FD)=M_{MF}\cdot F_{c}\cdot C_{kV}\cdot C_{FD}$$

To determine the maximum of the dose, we used more than one MOSFET dosimeter properly positioned and, as a final reading, we have taken the highest value. The possible dose underestimation due to the position of the detector has been taken into account in the overall uncertainty of MSD measurements.

### C. Overall MSD uncertainty and statistics

The overall uncertainty of the MSD measurements, Δ, was estimated as the sum of two contribution $\Delta_{1}$ and $\Delta_{2}$, where $\Delta_{1}$ represents the dose uncertainty associated with the accuracy of the dosimeter (i.e., calibration uncertainty, angular dependence, and fading) and $\Delta_{2}$ the uncertainty on the position of the detector. $\Delta_{1}$ was considered to be 10%, as reported in literature,^(29)^ while $\Delta_{2}$ was estimated to be equal to the maximum inhomogeneity (5%) measured with respect to the center of a $30\times 40\;{\text{cm}}^{2}$ field of view. The considered global uncertainty was therefore 15%.

Pearson's product‐moment correlations ($R_{p}$) were calculated to evaluate the relationship between the more readily available parameters (FT, Ka,r, and DAP values) and the less‐available quantity (MSD). Confidence intervals (CIs) were calculated using the *t*‐Student distribution. Spine procedures were considered all together; the CLI treatments were not included, being technically and procedurally too different from the other ones. Because of the small number of patients (ten) only in the CLI treatment, the correlations for this kind of procedure were not investigated. The results with a p‐value not greater than 0.05 were considered statistically significant.

## III. RESULTS

### A. Characterization and calibration procedures

**Figure 2:**
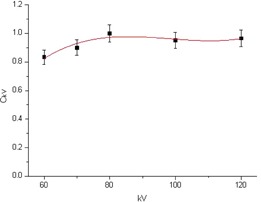
Energy correction factors $C_{\text{kV}}$ as a function of the tube voltage in reference conditions. A third‐grade polynomial fit was superimposed. The error propagation formula was used to calculate the error bars, taking into account the uncertainty of the device (4%) for the UNFORS, and the reproducibility at the corresponding dose value (4%) for the MOSFET reading.

A weak dependence of the dosimeter reading was observed as a function of the additional filters with a maximum variation of ±8%. Since this value was below the experimental uncertainties ($\Delta_{1}$) in the calibration process, we therefore decided to average the MOSFET readings measured in both filtering. The calibration factor value $F_{c}$ was set to 0.20. In Fig. 2, $C_{\text{kV}}$ data versus tube voltage inputs (60–120 kV) are shown. In order to allow the extrapolation of $C_{\text{kV}}$ at different tube voltages, a third‐order polynomial best‐fit function was chosen ($R^{2}$ value of 0.90). Finally, as the MOSFET readings with field size varied by at least 2% (well below the experimental uncertainties, $\Delta_{1}$), we decided to consider the field size correction factor $C_{\text{FD}}$ equal to 1 in all the investigated experimental conditions.

**Figure 3:**
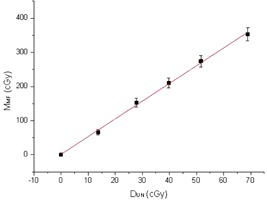
MOSFET reading in reference conditions vs. the dose measured by the reference detector. We considered the uncertainty of the device (4%) for the UNFORS, and the reproducibility at the corresponding dose value (4%) for the MOSFET reading. In this figure, only the error bars relative to MOSFET reading were shown.

Figure 3 displays the results of the MOSFET readings ($M_{\text{MF}}$) versus the dose measured with the reference detector ($D_{\text{UN}}$) in the RS and for doses up to 70 cGy. A linear dose dependence was found with a regression coefficient $R^{2}$ of 0.999.

The entrance surface dose in phantom measured using GAFCHROMIC XRRV3 films was found to be in agreement within their respective SDs both with the values measured by the reference detector and MOSFET detectors.

### B. Patient dose study

The spine procedures were all performed with pulsed fluoroscopy at 15 fps. In only the PVP procedures, variable‐frame radiography at 1 fps was used in some circumstances. The distance between the focus and image receptor was fixed to 120 cm. The PVP procedure, was carried out mainly in the LL projection (60% compared to 40% obtained in the AP projection); the gantry tilt ranged approximately from 0° to 20°. The PDD, instead, was on average performed 70% in LL and 30% in AP; the tilt angle ranged between $\pm 20^{\circ }$. In the RF procedure, finally, a fixed rotation angle of the tube with respect to the couch axis was used, which ranged from $-30^{\circ }$ to $+30^{\circ }$, depending on the patient. In such procedure, the tilt angle was negligible. For each intervention, in the case of PDD, only one vertebral level was treated; for PVP and RF procedures, on average, four vertebral levels and eight vertebral facets were treated, respectively.

The CLI procedures used both pulsed fluoroscopy at 15 fps and variable‐frames radiography at 1 fps (about 90% compared to 10%, respectively). DSA (Digital Substraction Angiography) of the vessel below the knee was used by employing a contrast medium of about 7 ml of injectable solution of iobitridol (XENETIX 300 mg/ml, Guerbet S.p.a., Villepinte, France). The tilt and rotation angle of the tube were found to vary from $+5^{\circ }$ to $-5^{\circ }$ and from $+30^{\circ }$ to $-40^{\circ }$, respectively, being more pronounced at the foot and ankle level with respect to the femur and knee. The mean distance between the focus and the image detector was 90 cm.

The mean dosimetric data, calculated over ten patients for the spine procedures and for the CLI treatment, are shown in Table 1 and Table 2, respectively. The tables report patients’ age, gantry position, time‐weighted average of the tube voltage and current values in fluoroscopy modality (both for each gantry position and cumulative for the two projections), cumulative fluoroscopy time, number of images in variable‐frame radiography, local maximum skin dose, cumulative air kerma, and cumulative dose‐area product. The values reported in brackets represent the range of variation. In Table 2, the values of the parameters are reported for different regions of the body: femur, knee, and ankle.

The PVP procedure recorded the highest average MSD (in LL position), Ka,r, and DAP values: 10.8 cGy, 114.6 Gy•cm^2^ and 1106.4 mGy, respectively. On the contrary, the CLI treatments showed the lowest MSD value (2.3 cGy), the PDD the lowest DAP (25.4 Gy•cm^2^), and RF the lowest Ka,r value (306.3 mGy). It is worth noting that CLI treatments registered the longest fluoroscopic times and the highest number of radiographic images, but the current values employed in this kind of treatments were significantly lower.

**Table 1:** Measured data for spine interventional procedures on 30 patients (10 for procedure)

			*Fluoroscopy*								
*Procedure*	*Patients’ Mean Age (y)*	*Gantry Position (°)*	*Tube Voltage (kV)*	*Current (mA)*	*Cumulative Tube Voltage (kV)*	*Cumulative Current (mA)*	*Cumulative Fluoroscopy Time (min)*	*Number of Radiographic Images*	*MSD (cGy)*	*Ka,r (mGy)*	*DAP (Gy·cm^2^)*
PVP	70	AP	90 (71‐120)	14 (13‐16)	103 (71‐120)	16 (13‐20)	17.2 (6.8‐25.2)	0	7.5±1.1 (0.4‐47.1)	1106.4 (402‐2610)	114.6 (42.2‐240.5)
		LL	107 (85‐120)	17 (14‐20)				2	10.8±1.6 (0.8‐54.1)		
PDD	56	AP	95 (85‐120)	15 (14‐16)	110 (85‐120)	16 (14‐19)	4.6 (1.9‐13.2)	0	3.6±0.5 (0.3‐13.2)	442.5 (96.3‐1695.5)	25.4 (11.8‐49.5)
		LL	115 (90‐120)	17 (14‐19)				0	9.0±1.4 (0.7‐33.1)		
RF	74	AP			108 (80‐120)	16 (15‐18)	5.9 (2‐9.1)	0	4.2±0.6 (1.0‐11.0)	306.3 (125‐554.3)	30.5 (14.1‐53.3)

PVP=percutaneous vertebroplasty; PDD=percutaneous disc decompression; RF=radiofrequency medial branch neurolysis; MSD=maximum skin dose; Ka,r=air kerma; DAP=dose‐area product.

**Figure 4:**
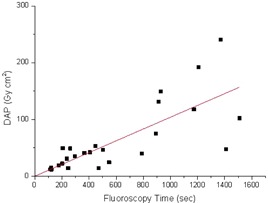
Measured DAP for all the investigated spine interventional procedures as a function of the fluoroscopy time. The continuous line represents the linear best fit.

**Figure 5:**
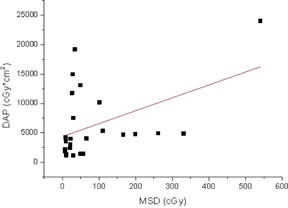
Measured DAP for all the investigated spine interventional procedures as a function of the MSD. The continuous line represents the linear best fit.

**Figure 6:**
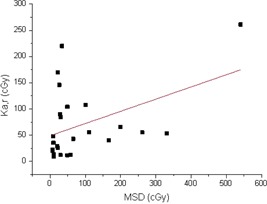
Measured Ka,r for all the investigated spine interventional procedures as a function of the MSD. The continuous line represents the linear best fit.

The Pearson's correlation coefficients among the FT, Ka,r, DAP. and MSD are displayed in Table 3. FT and DAP showed a statistically significant correlation ($R_{p}=0.76,p<0.01$, two‐tailed *t*‐test). The DAP and MSD, such as the Ka,r and MSD values, showed a worse correlation ($R_{p}=0.46,p<0.05$ and $R_{p}=0.40,p<0.03$, respectively, using the two‐tailed *t*‐test). The scatter plots of the DAP as a function of the fluoroscopy time, the Ka,r as a function of MSD, and DAP as a function of MSD are displayed in Figs. 4, 5, and 6, respectively. These figures clearly emphasize that it is not possible to estimate DAP from FT, or Ka,r from MSD, or DAP from MSD with an acceptable accuracy.

Finally, for the FT and MSD, a not statistically significant correlation ($R_{p}=0.29,p=0.16$, two‐tailed *t*‐test) was observed.

**Table 2:** Measured data for critical limb ischemia treatments on 10 patients

	*Fluoroscopy*		*Radiography*						
*Irradiated Anatomical area*	*Kilovoltage (kV)*	*Current (mA)*	*Kilovoltage (kV)*	*Current (mAs)*	*Cumulative fluoroscopy time (min)*	*Number of radiographic images*	*MSD (cGy)*		*Ka,r (mGy)*	*DAP (Gy·cm²)*
FEMUR	67 (60‐81)	11 (7‐21)	72 (65‐80)	10 (7‐18)			${\text{CLI}}_{\text{PF}}$	0.5±0.1 (0.2‐1.0)		
KNEE	65 (58‐75)	10 (6‐15)	72 (65‐80)	9 (5‐12)	41.1 (14.5‐139.6)	125	${\text{CLI}}_{\text{DF}}$	2.3±0.3 (0.2‐4.1)	485.8 (112.9‐2160)	74.6 (29.9‐245.0)
							${\text{CLI}}_{\text{PT}}$	1.8±0.3 (0.8‐4.8)		
ANKLE	64 (52‐70)	7,5 (5‐12)	72 (65‐80)	5 (2‐9)			${\text{CLI}}_{A}$	1.2±0.2 (0.3‐2.6)		

${\text{CLI}}_{\text{PF}}=\text{measurements on the proximal third femur}$; ${\text{CLI}}_{\text{DF}}=\text{measurements on the distal third femur}$; ${\text{CLI}}_{\text{PT}}=\text{measurements on the proximal third tibia}$; ${\text{CLI}}_{A}=\text{measurements on the lower part of the ankle}$; MSD=maximum skin dose; Ka,r=air kerma; DAP=dose‐area product.

**Table 3:** $R_{p}$ coefficients (95% CI) and p‐values for the Pearson's test

	$R_{p}$	*p‐value*
*FT‐DAP*	0.76	<0.01
*FT‐MSD*	0.29	0.16
*Ka,r‐MSD*	0.40	<0.03
*DAP‐MSD*	0.46	<0.05

FT−DAP=fluoroscopy time−dose‐area product; FT−MSD=fluoroscopy time−maximum skin dose; Ka,r−MSD=air kerma−maximum skin dose; DAP−MSD=dose‐area product−maximum skin dose; Rp=Pearson's product‐moment correlation.

## IV. DISCUSSION

The cable‐free OneDose system has been calibrated in kV energy range and in water‐equivalent phantom in order to determine, in a reference setting, the response of the dosimeters ($F_{c}$) and how it varies with X‐ray energy ($C_{\text{kV}}$) and field size ($C_{\text{FD}}$). As it was found to be slightly dependent on the additional Cu filters (within the experimental uncertainty), the MOSFET readings were averaged on the used filtrations. An $F_{c}$ value of 0.20 was found which corresponds to a response of the dosimeter in the kV energy range five times greater than in the MV energy range. This is in agreement with the value reported in literature,^(29)^ using the same methodology and detectors. In addition, such calibration was confirmed by the comparison performed with GAFCHROMIC films. However, some differences in the behavior of the $C_{\text{kV}}$ were observed,^(29)^ indicating that the calibration procedure is strongly dependent on the X‐ray system used.

The dosimetric data of the FGIs on the patients are summarized in Tables 1 and 2. From Table 1 it can be noticed that both PVP and PDD procedures registered MSD values measured in AP projection lower than those measured in LL projection (40% and 25% for AP and 60% and 75% for LL, respectively), according to the corresponding percentages of fluoroscopy time on the X‐ray tube views. The PVP procedure registered much higher average Ka,r and DAP values than those of PDD and RF. This is clearly due to the much longer fluoroscopy time employed for such procedures with respect to the others. Similarly, a much higher MSD value for the PVP with respect to the RF was observed. However, despite the much shorter fluoroscopy time, Ka,r and DAP, the PDD procedure led to a MSD value comparable to that of PVP (9.0 cGy and 10.8 cGy, respectively). This can be explained by observing that in the PVP and RF procedures, more vertebral levels were treated for a single patient. Consequently, the X‐ray beam moved along different portions of skin above the treated vertebrae, thus delivering a lower local dose. In the PDD procedures, instead, the irradiation was focused on a single vertebra and in LL projection, delivering all the dose to a small skin region.

In CLI procedures, a much lower MSD value (2.3 cGy) was registered as compared to the spine procedures, even though the cumulative FT and the number of radiography images were considerably higher. In such procedures, the average mA, mAs, and kV values were lower, reducing the dose absorbed by the skin. Moreover, in the CLI procedure the radiation was spread out from the femur to ankle in the same manner as discussed above for RF.

From the statistical analysis, both Ka,r and DAP have been found to have a poor correlation with MSD. Moreover, the corresponding scatter plots of Figs. 5 and 6 indicate that it is impossible to predict MSD from DAP or Ka,r with an acceptable accuracy ($R^{2}=0.21$ and $R^{2}=0.19$, respectively), as also reported in literature.^12^, ^30^, ^31^, ^32^ Therefore, to quantify the deterministic effects, a direct measure of MSD is recommended. DAP correlated better with FT than with MSD, even though a prediction of DAP from FT can again lead to error of the order of ±50%, as it can be seen from Fig. 4.

Indeed, as reported by Jones and Pasciak,^(33)^ it is possible to estimate the MSD using dose metrics such as the Ka,r, DAP, or other data that are commonly available on modern fluoroscopes. However, additional specific steps must be followed to convert such dose metrics into MSD. In particular, some factors that may affect actual MSD, such as the attenuation of couch, the f‐factor, the backscatter factor, the half value layer, and patient thickness have to be taken into account. As a consequence, the determination of the MSD following this method is relatively complex, and the above‐mentioned factors have to be known with high precision. Therefore, the feasibility of such a method strongly depends on the complexity of the treatment and on the data available on the fluoroscope. In any case, a comparison with physical measurements is recommended to verify the accuracy of this technique.

Table 4 shows a comparison between our data with those reported in literature.^32^, ^34^, ^35^, ^36^, ^37^, ^38^, ^39^, ^40^, ^41^

For PVP procedures, we registered a DAP value higher than those reported in literature. On the contrary, we measured MSD of one smaller magnitude order. These differences can be probably due to different X‐ray field sizes used during the procedure (unknown for the data collected in literature), or to the number of treated vertebrae. However, it is noteworthy that D'Ercole et al.^(37)^ reported MSD values for AP an LL separately, with a ratio comparable with ours (40% in AP with respect to 60% in LL).

For the RF procedures, Acho et al.^(38)^ measured a MSD of about a third of our value and a much shorter fluoroscopy time. Similarly, Manchikanti et al.^(39)^ reported a very brief fluoroscopy time. Such difference can be ascribed to the different number of vertebral facets treated for each intervention (eight vertebral facets, on average, in our case).

With regard to the PDD procedure, to our knowledge no dosimetric data are available in literature.

Concerning CLI treatments, the dosimetric data are deeply dependent on the patient and clinical complexity. We registered a higher DAP and a lower MSD than the values reported by Bor et al.^(40)^ which used only 2.2 min of fluoroscopy time (diagnostic only). The Bor study concentrated radiations mainly in the pelvic region, probably requiring more mAs than those used in our treatments. On the other hand, our procedure was at the same time both diagnostic, for below‐the‐knee procedures, and therapeutic. In particular, it focused on the knee and ankle as well, where the lower thickness involved required fewer mAs. Kruger et al.^(41)^ registered on average higher DAP and fluoroscopy time values lower than ours, even though he declared an uncertainty of 150% on the DAP measurement.

**Table 4:** Comparison between our data and those reported in the literature for the investigated procedures

			*Fluoroscopy*					*MSD*	
*Procedure*	*Reference*	*Pts*.	*Cumulative Kilovoltage (kV)*	*Cumulative Current (mA)*	*Cumulative Fluoroscopy Time (min)*	*No. of Radiographic Images*	*DAP (Gy·cm^2^)*	*(cGy) AP*	*(cGy) LL*
PVP	34	10	(90‐100)	(6‐15)	10.4 (3‐28)	*‐*	41.0 (10.5‐117.0)	*‐*	*‐*
	35	11	(80‐110)	(2.5‐4.5)	27.7 (18.8‐43.1)	*‐*	‐		68.8±21.8 (44.6‐103.4)
	36	10	‐	‐	16.5 (10‐34)	*‐*	54 (14‐133)	*‐*	‐
	32	61	‐	‐	17.4 (4.2‐54)	82	77.6 (6.6–335.3)		68.4^b^ (7.8‐218.3)
	37	16	‐	‐	‐	‐	‐	69.0±44.7 (18.4‐183.4)	117.9±56.3 (41.7‐236.2)
	This work	10	103^a^ (71‐120)	16^a^ (13‐20)	17.2 (6.8‐25.2)	2	114.6 (42.2‐240.5)	7.5±1.1 (0.4‐47.1)	10.8±1.6 (0.84‐54.1)
RF	38	3	63.3 (54‐93)	‐	0.192 (1.4s‐30.0s)	‐	‐	1.4 (0.6‐2.7)	
	39	16	‐	‐	0.212 (6s‐23s)	‐	‐	‐	
	This work	10	101^a^ (80‐120)	16^a^ (15‐18)	5.9 (2‐9.1)	0	30.5 (14.1‐53.3)	4.2±0.6 (1.0‐11.0)	
CLI	41^c^	8	69 (54–78)	‐	2.2 (0.1–6.3)	36	18 (0.2–43)	3.6 (0.1–15.9)	
	40	96	‐	‐	16 (2.8–51.1)	‐	140.5 (5.9‐1506.4)	‐	
	This work	10	65^a^ (52‐81)	9.5^a^ (5‐21)	41.1 (14.5‐139.6)	125	74.6 (29.9‐245.0)	2.3±0.3 (0.8‐4.8)	

a Values averaged, for each part of the body under investigation, over 10 patients.

^b^ Peak skin dose (PSD) defined as the highest air kerma at any portion of a patient's skin during a procedure.

^c^ The values refer to therapeutic procedure.

MSD=maximum skin dose; DAP=dose‐area product; PVP=percutaneous vertebroplasty, RF=radiofrequency medial branch neurolysis; CLI=critical limb ischemia.

In literature, the RLs are proposed for only PVP and CLI treatments. For PVP, in terms of DAP and fluoroscopy time, our values are comparable to the RLs suggested by Miller et al.^(9)^ for the U.S. practice (i.e., a mean DAP value of 114.6 Gy•cm^2^ vs. 120 Gy•cm^2^ and a mean fluoroscopy time of 17.2 min vs. 21 min, respectively). As for CLI treatments, some authors propose national reference levels for lower limb angiography.^8^, ^10^, ^42^, ^43^, ^44^ Only Vano et al.^(42)^ provide values of preliminary RLs (in terms of DAP and fluoroscopy time) both for diagnostic and peripheral therapeutic procedures, separately. In our case, both the angiography and the angioplasty were performed during the same intervention so that the measured dosimetric values were compared to the sum of the RL values given in literature.^(42)^ The fluoroscopy time observed for CLI procedures was sensibly higher than the RL value reported in literature.^(42)^ However, our value of DAP and the number of radiographic images were much smaller than the proposed reference level (140 Gy•cm^2^ and 500 frames). The measured DAP value was smaller than the reference level, despite the relatively long fluoroscopy time, thanks to the much lower number of radiographic images which are known to increase the dose delivered to the patient.

In all the investigated procedures, the registered MSD values were far below the threshold value for the deterministic effects (2 Gy for early transient erythema) and, therefore, they can be considered safe for the patients. However, in some cases, these procedures could be repeated over the time, as well, as new interventions on vertebrae close to those already treated. These circumstances increase the probability of stochastic effects and, in the more severe cases, the dose can approach the threshold for deterministic effects. However, these events are extremely rare and no patients reported skin injuries due to radiation in our institute.

A further study, based on a larger number of patients and extended over more hospitals with different X‐ray equipments, could strengthen the results of the statistical analysis and allow a more proper RLs evaluation.

## V. CONCLUSIONS

The interventional procedures investigated are both safe with regard to deterministic effects and optimized for stochastic ones. However, particular care has to be devoted to those patients who could need more than one intervention. For MSD measurements, the MOSFET‐based system was found to be suitable and comfortable both for the patient and operator, being cable free and able to provide fast readings of the dose absorbed by the skin. Finally, the observed correlations between DAP and FT indicated that the estimation of the DAP from FT can be feasible in the spine procedures, but is not very precise. On the other hand, poor correlation between the measured MSD and Ka,r, DAP or FT was observed so that a direct measure of MSD is, therefore, recommended.
